# α-Lipoic Acid Alleviates Non-Alcoholic Fatty Liver Disease by Elevating Chaperone-Mediated Autophagy and Increasing β-Oxidation via AMPK-TFEB Axis

**DOI:** 10.3390/nu18030402

**Published:** 2026-01-26

**Authors:** Keting Dong, Miao Zhang, Jiaojiao Xu, Xue Bai, Jianhong Yang

**Affiliations:** Medical School, University of Chinese Academy of Sciences, Beijing 101400, China; dongketing21@mails.ucas.ac.cn (K.D.);

**Keywords:** non-alcoholic fatty liver disease (NAFLD), α-lipoic acid (ALA), transcription factor EB (TFEB), chaperone-mediated autophagy (CMA), nuclear factor-erythroid 2-p45 derived factor 2 (NRF2)

## Abstract

Background: Non-alcoholic fatty liver disease (NAFLD) is a prevalent chronic liver disorder associated with impaired lipid metabolism and oxidative stress. As a natural antioxidant and dithiol compound, α-lipoic acid (ALA) may play a beneficial role in modulating hepatic metabolism. This study investigates the potential mechanisms through which ALA may alleviate NAFLD. Methods: To construct an NAFLD model, NCTC 1469 cells were exposed to oleic acid and palmitic acid (OA/PA) and glucose for 24 h. RT-qPCR, Western blotting, and siRNA analyses were used to examine the effects and mechanisms of ALA. In vivo, C57BL/6J mice were fed a high-fat diet for 11 weeks and treated with ALA (200 mg/kg/day, intragastrical) for 4 weeks to evaluate its impact on NAFLD. Results: In NCTC 1469 cells exposed to OA/PA and glucose, ALA markedly reduced lipid accumulation by activating TFEB, which in turn promoted fatty acid β-oxidation and chaperone-mediated autophagy (CMA). Furthermore, ALA activated NRF2-dependent CMA and mitigated oxidative stress. Inhibition of AMPK or silencing of TFEB/NRF2 abolished these effects, indicating the key role of the AMPK–TFEB/NRF2 axis. In HFD-fed mice, ALA alleviated hepatic steatosis, serum lipid abnormalities, and liver injury, consistent with its activation of CMA and β-oxidation and reduction in oxidative stress via this pathway. Conclusions: ALA synchronously activates CMA, β-oxidation, and antioxidant responses via a unified AMPK pathway to reduce lipid accumulation and oxidative stress, providing a mechanistically integrated therapeutic strategy for NAFLD.

## 1. Introduction

Non-alcoholic fatty liver disease (NAFLD) is defined by abnormal lipid deposition within hepatocytes without excessive alcohol intake [1]. Its clinical progression encompasses a broad spectrum, ranging from simple steatosis and non-alcoholic steatohepatitis to advanced liver cirrhosis and hepatocellular carcinoma [2,3]. Although NAFLD has recently been re-termed metabolic dysfunction-associated steatotic liver disease (MASLD) in clinical settings, the term NAFLD is still widely used in preclinical studies due to differences in diagnostic criteria and model applicability.

From a mechanistic perspective, hepatic lipid accumulation is caused by an imbalance between the input and clearance of fatty acids. Impaired mitochondrial β-oxidation and the dysregulation of chaperone-mediated autophagy (CMA) reduce the capacity for fatty acid catabolism and lipid turnover. This contributes to abnormal lipid accumulation. Emerging studies suggest that the modulation of autophagy and oxidative stress may contribute to the amelioration of NAFLD as part of a multifactorial therapeutic strategy [4,5,6].

Among the regulators involved in these processes, transcription factor EB (TFEB) has been implicated in the regulation of hepatic lipid metabolism. TFEB activation is markedly negatively correlated with the extent of hepatic steatosis in patients with NAFLD [7]. Mechanistically, TFEB promotes fatty acid β-oxidation by activating peroxisome proliferator-activated receptor α (PPARα) and its coactivator PPAR-γ co-activator-1 α (PGC1α) and enhances CMA by regulating lysosomal-associated membrane protein 2 (LAMP2A), thereby exerting a global transcriptional control on lipid catabolism [8,9,10].

β-Oxidation is the main type of fatty acid oxidation. It is predominantly facilitated within the mitochondria and is regulated by PPARα [11]. Research has found that Formononetin alleviates NAFLD by activating the PGC-1α/PPARα signaling pathway, enhancing β-oxidation and thereby reducing lipid accumulation [12].

CMA is a lysosomal degradation pathway. In this pathway, the heat shock cognate 71 kDa protein (HSC70) selectively binds to substrate proteins that possess the KFERQ pentapeptide motif, which facilitates their translocation across the lysosomal membranes into the lysosomal lumen through interactions with LAMP2A [13]. Recent studies suggest that dysregulated CMA contributes to an imbalance in hepatic lipid metabolism. Impaired CMA disrupts the degradation of PLIN5, resulting in defective lipid droplet turnover in NAFLD models [14]. It also suppresses fatty acid oxidation by accumulating NCoR1, which exacerbates hepatic steatosis [15]. Moreover, emerging studies link CMA dysfunction to NASH progression via enhanced cholesterol accumulation and endoplasmic reticulum stress [16]. Taken together, these findings emphasize the importance of CMA as a metabolic regulator in NAFLD and related metabolic liver diseases [17].

Given the central role of CMA in hepatic lipid homeostasis, increasing attention has been directed toward identifying upstream regulatory pathways that modulate CMA activity. Nuclear factor-erythroid 2-p45 derived factor 2 (NRF2) is a master regulator of antioxidant responses and has recently been implicated in CMA regulation. Increasing evidence suggests that NRF2 directly activates LAMP2A transcription, thereby enhancing CMA activity while simultaneously alleviating oxidative stress [18,19,20,21].

In this context, AMP-activated protein kinase (AMPK), a key regulator of energy homeostasis, has been associated with metabolic disorders, including diabetes, obesity, cancer, and fatty liver diseases. Activation of AMPK has been shown to alleviate NAFLD [22]. Previous studies demonstrated that buddleoside alleviates NAFLD by targeting the AMPK-TFEB pathway [23], while α-Ketoglutarate prevents fatty liver by activating the AMPK-PGC1α/NRF2 pathway [24].

α-lipoic acid (ALA) is a naturally occurring antioxidant and mitochondrial coenzyme widely used in the treatment of metabolic and inflammatory disorders [25,26,27,28,29,30,31,32,33]. Longhitano et al. demonstrated that ALA improves NAFLD in HepG2 cells by modulating mitochondrial homeostasis, oxidative stress, and partial genes involved in lipid metabolism [34,35,36], while Sztolsztener et al. showed that ALA improves NAFLD in Wistar rats by regulating sphingolipid metabolism and insulin transduction [37]. Although previous studies indicate the partial mechanisms responsible for the effects of ALA on NAFLD, the molecular mechanisms regulating lipid metabolism have yet to be fully characterized.

In the present study, we aimed to investigate the protective effects of ALA against hepatic steatosis and to explore the underlying molecular mechanisms. Specifically, we examined whether ALA modulates chaperone-mediated autophagy, fatty acid β-oxidation, and oxidative stress through AMPK-dependent signaling pathways, including TFEB and NRF2, in both in vitro and in vivo models of NAFLD. By addressing these questions, this study seeks to provide new mechanistic insights into the therapeutic potential of ALA in NAFLD.

## 2. Materials and Methods

### 2.1. Cells and Cell Culture

The normal mouse hepatocyte cell line NCTC 1469 was purchased from Procell Life Science & Technology (Wuhan, China), and the cells were cultivated in DMEM (Procell) containing 10% donor equine serum (HS, Procell). The cells were then cultured in a humid incubator at 37 °C, with 5% CO_2_. For the treatment, a hepatocyte steatosis model was induced with 250 μM oleic acid and 125 μM palmitic acid (250 μM OA/125 μM PA), as well as 60 mM glucose with or without 200 μM α-lipoic acid (ALA; IL0180; Solarbio; Beijing, China) for 24 h. In the AMPK inhibition assay, cells at approximately 80% confluence were preincubated with 20 μM dorsomorphin (Compound C, CpdC; HY-13418A; MedChemExpress; Shanghai, China) for 1 h prior to subsequent treatment.

### 2.2. Animals and Treatment

Male C57BL/6J mice aged six weeks were sourced from Beijing Vital River Laboratory Animal Technology Co., Ltd. (Beijing, China). After a one-week acclimation period, mice were randomly assigned to either a normal chow diet group (NCD; BEIJING KEAO XIELI FEED Co., Ltd.; Beijing, China) or a high-fat diet group (HFD; Dyets; Wuxi, China). After 11 weeks of HFD feeding, mice in the HFD group were further randomly allocated to either an HFD group receiving vehicle treatment (*n* = 4; sodium carboxymethyl cellulose solution, CMC-Na; IS9000; Solarbio) or an ALA treatment group (*n* = 4; D8761; Solarbio). Mice in both HFD groups were maintained on the HFD for a total of 15 weeks. Because CMC-Na can both suspend ALA in solution and form a protective coating to prevent its oxidation, ALA was prepared as a CMC-Na suspension and administered orally via a 10 μL gavage needle. According to prior studies indicating that 200 mg/kg ALA is the most commonly used dose and the most effective for liver protection, the mice received ALA at 200 mg/kg once per day. All mice were kept in a specific barrier environment under controlled conditions, including a 12-h light/dark cycle, ad libitum access to food and water, and a stable ambient temperature of 24 ± 1 °C. Following four weeks of treatment, the mice were anesthetized with 1% pentobarbital sodium (50 mg/kg). Then, approximately 500 μL of blood was collected from the eyeball of each mouse, after which they were humanely sacrificed by cervical dislocation. The serum and liver were then collected for the detection of relevant indicators. All animal procedures and group allocations were conducted in accordance with the approved institutional ethical guidelines.

### 2.3. Cell Viability Assay

NCTC 1469 cells were inoculated into 1 × 10^4^ cells per well in 96-well plates. After a 24 h culture period, the cells were subjected to stimulation as outlined in Section 2.1. The cells were incubated with CCK8 for a further 1 h and cell viability was analyzed at a wavelength of 450 nm.

### 2.4. Cell Staining

After treatment, the cells were gently rinsed with PBS and subsequently fixed in 4% paraformaldehyde (Leagene; Beijing, China) for 15 min. Lipid staining was carried out by incubating the samples with 4 µM BODIPY 493/503 (GC42959; GLPBIO; Shanghai, China) for 30 min or with Oil Red O (0.3%, *w*/*v*, O8010, Solarbio) for 10 min, both performed at room temperature. Following staining, residual dye was thoroughly removed by repeated PBS washes. Finally, stained cells were visualized and photographed under a Leica Microsystems optical microscope (Wetzlar, Germany) at 200× magnification. All stained images were quantified using ImageJ version 6. For each experimental condition, images were acquired from randomly selected fields under identical microscope settings. At least 3 independent fields per well were analyzed, and the mean value was calculated for statistical analysis. Quantification was performed by fluorescence intensity according to the actual method used.

### 2.5. Quantitative Real-Time PCR

After cell processing, total RNA was extracted using TRIzol (BS2594, Biosharp; Beijing, China). One microgram of total mRNA was then reverse-transcribed into cDNA using the Hifair III SuperMix (11141ES60, Yeasen; Shanghai, China). Then, the cDNA was amplified using Top Green qPCR SuperMix (AQ132-24; TransGen Biotech; Beijing, China). The primer sequences are detailed in Table 1. Gene expression analysis was performed using the 2^−ΔΔCt^ method.

### 2.6. TFEB and NRF2 Knockdown

SiRNAs specific to mouse TFEB (siRNA-775: sense, CCCCGAGAAAGAGUUUGAUTT, and antisense, AUCAAACUCUUUCUCGGGGTT; siRNA-1994: sense, CAGAAGAGAAAUAAGAAGATT, and antisense, UCUUCUUAUUUCUCUUCUGTT; siRNA-2296: sense, UCCCACUUUGUGCCUUUAGTT, and antisense, CUAAAGGCACAAAGUGGGATT) and non-targeting control siRNA were obtained from Sangon Biotech (Shanghai, China).

SiRNAs targeting mouse NRF2 (siRNA-334: sense, GGAUGAAGAAACAGGAGAATT, and antisense, UUCUCCUGUUUCUUCAUCCTT; siRNA-668: sense, GAAUUACAGUGUCUUAAUATT, and antisense, UAUUAAGACACUGUAAUUCTT; siRNA-1720: sense, CGACAGAAACCUCCAUCUATT, an-d antisense, UAGAUGGAGGUUUCUGUCGTT) and non-targeting control siRNA were obtained from Sangon Biotech.

SiRNA transfection into NCTC 1469 cells was performed using the Liposomal 2000 Transfection Reagent (40802ES03, Yeasen) in accordance with the protocol provided by the manufacturer.

### 2.7. Biochemical Analysis

Biochemical parameters, including triglyceride (TG), total cholesterol (TC), aspartate aminotransferase (AST), alanine aminotransferase (ALT), superoxide dismutase (SOD) activity, and malondialdehyde (MDA) content, were determined according to the manufacturers’ instructions using commercially available assay kits. TG (A110-1-1), TC (A111-1-1), AST (C009-2-1), and ALT (C010-2-1) kits were purchased from Nanjing Jiancheng Bioengineering Institute (Nanjing, China), while SOD (BC0175) and MDA (BC0025) kits were obtained from Solarbio (Beijing, China).

### 2.8. Western Blotting

NCTC 1469 cells were subjected to treatment, after which, total cellular proteins were isolated using RIPA lysis buffer (R0010; Solarbio) following the standard protocol. The lysates were treated with ultrasound on ice at 20% amplitude for five seconds per cycle, repeated three times, to ensure complete cell disruption. After centrifugation at 12,000 rpm for 10 min, the clarified supernatant was collected for protein analysis. Protein concentrations were determined using a BCA protein assay kit (B5001; LABLEAD; Beijing, China). Equal amounts of protein (40 µg) were then resolved by SDS-PAGE and transferred onto polyvinyl difluoride (PVDF) membranes.

Membranes were blocked with 5% non-fat dry milk (D8340; Solarbio) or bovine serum albumin (BSA, A6020A; Biotopped; Beijing, China) prepared in TBST for two hours at room temperature and subsequently incubated overnight at 4 °C with the appropriate primary antibodies. Primary antibodies purchased from Cell Signaling Technology (Danvers, MA, USA) were anti-AMPKα (D5A2) rabbit mAb (1:2000; 5831), anti-Phospho-AMPKα (Thr172) (40H9) Rabbit mAb (1:2000; 2535) and SOD2 (1:2000; D3X8F) XP^®^ Rabbit mAb. Primary antibodies obtained from Proteintech Group (Chicago, IL, USA) were: TFEB polyclonal antibody (1:2000; 13372-1-AP), PGC1α monoclonal antibody (1:2000; 66369-1-Ig), Perilipin-2 polyclonal antibody (1:2000; 15294-1-AP), NRF2, NFE2L2 polyclonal antibody (1:2000; 16396-1-AP), and β-actin recombinant antibody (1:2000; 81115-1-RR). Primary antibodies against anti-PPARα rabbit pAb (1:500; WL00978) and anti-NQO1 rabbit pAb (1:500; WL04860) were purchased from Wanleibio (Wuhan, China). Primary antibodies against anti-LAMP2 rabbit pAb (1:1000; DF6719), anti-HSC70 rabbit pAb (1:2000; AF5187) and anti-HO-1 rabbit pAb (1:2000; AF5393) were purchased from Affinity Biosciences (Liyang, China). Following three washes with TBST (10 min each), membranes were incubated with HRP-conjugated secondary antibodies (S0101 and S0100; LABLEAD Biotech) for one hour at room temperature. For second development, after treatment with stripping buffer (P1652, APPLYGEN; Beijing, China), the membranes were re-incubated with the indicated primary antibodies. Band intensities were quantified from corresponding molecular weight regions and normalized to ACTB. Identical exposure settings were applied to all samples within each experiment. Protein signals were detected using an enhanced chemiluminescence (ECL, 170-5060; Bio-Rad, Hercules, CA, USA) system, and band intensities were quantified using ImageJ version 6.

### 2.9. Immunofluorescence

Following the specified treatments, the cells were gently rinsed three times with cold PBS and fixed using 4% paraformaldehyde (DF0135; Leagene). After fixation, the cells were treated with proteinase K for 10 min, after which they were washed with PBS. The samples were then blocked with 5% goat serum and incubated with the primary antibodies at 4 °C overnight. After washing, the cells were incubated with a fluorescently labeled secondary antibody (1:200) in the dark for 1 h. The nuclei were then counterstained with DAPI, and fluorescence images were acquired using a laser scanning confocal microscope.

### 2.10. Measurement of CMA Activity Using PA-mCherry-KFERQ Reporter

NCTC 1469 cells were cultured in 15 mm glass-bottom dishes and transduced with pSIN-PAmCherry-KFERQ-NE lentiviral particles (plasmid #102365 Addgene, Watertown, MA, USA). Photoactivation was performed using 405 nm UV-A light for 10 min. Sixteen hours after photoactivation, cells were fixed with 4% paraformaldehyde and analyzed by confocal microscopy. Quantification was performed by counting the number of red fluorescent puncta per cell using ImageJ software.

### 2.11. Detection of ROS Content by Flow Cytometry

Intracellular reactive oxygen species (ROS) levels were measured by flow cytometry using 2′,7′-dichlorofluorescein diacetate (DCFH-DA; CA1410; Solarbio). After the indicated treatments, cells were harvested by trypsinization, washed three times with PBS, and incubated with 10 μM DCFH-DA at 37 °C for 30 min in the dark. Cells were then washed three times with PBS to remove excess probe, resuspended in PBS, and analyzed using a flow cytometer. Data were processed using FlowJo software version 10.10.0.

### 2.12. Statistical Analysis

Statistical analysis of the data was carried out using GraphPad Prism (version 9.0). Data from at least three independent experiments are expressed as the mean ± SD. Statistical differences between two groups were evaluated using Student’s *t*-test, whereas multiple group comparisons were assessed by one-way ANOVA followed by Tukey’s post hoc test. A *p*-value of less than 0.05 was considered to indicate statistical significance.

## 3. Results

### 3.1. ALA Suppresses the Accumulation of Lipids in NCTC 1469 Cells Induced by HGHF (OA/PA Combined with 60 mM Glucose)

**Figure 1:**
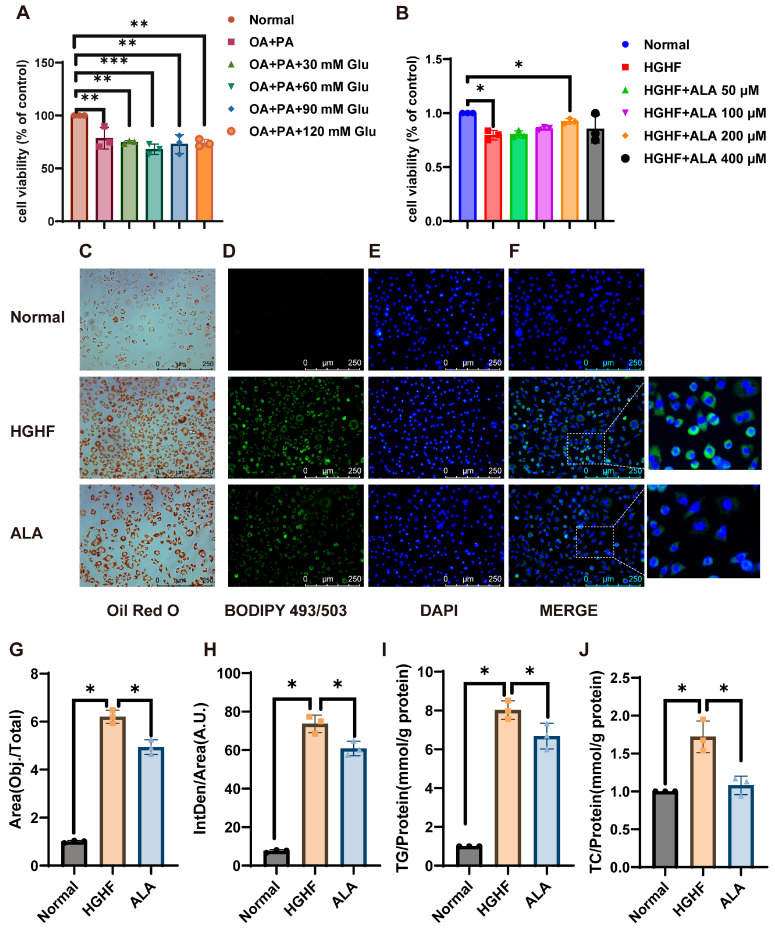
ALA suppresses the accumulation of lipids in cells induced by HGHF. (**A**,**B**) Cell viability was assessed using the CCK-8 assay; data represent the results of three independent experiments. (**C**) Representative images of cells stained with Oil Red O (200×). (**D**–**F**) Representative fluorescence images showing BODIPY 493/503 staining (green) of lipid droplets, with nuclei counterstained with DAPI (blue). (**G**,**H**) Mean fluorescence intensity of (**C**,**D**). (**I**) Intracellular TG levels in NCTC 1469 cells were measured using a triglyceride assay kit following the manufacturer’s protocol. (**J**) Intracellular TC levels in NCTC 1469 cells were measured using a total cholesterol assay kit following the manufacturer’s protocol. * *p* < 0.05, ** *p* < 0.01, *** *p* < 0.001.

To set up a hepatic lipid accumulation model, NCTC 1469 cells were treated with glucose (0–120 mM) combined with OA/PA. Treatment with OA/PA alone or in combination with glucose resulted in decreased viability, and OA/PA combined with 60 mM glucose was selected as the optimal model group due to its stable effects (Figure 1A). Subsequent experiments identified 200 µM ALA as the optimal dose for mitigating OA/PA combined with 60 mM glucose-induced viability loss (Figure 1B).

Then, we assessed lipid accumulation using Oil Red O and BODIPY 493/503 staining, as well as TG and TC quantification. The OA/PA combined with 60 mM glucose group exhibited significantly elevated lipid levels compared to normal groups (Figure 1C–H), confirming successful model induction. ALA treatment markedly reduced lipid deposition, further validated by decreased TG and TC levels (Figure 1I,J). Therefore, in subsequent experiments, cells treated with OA/PA combined with 60 mM glucose were designated as the high-glucose high-fat (HGHF) group, while those receiving ALA treatment were defined as the ALA group. These results indicate ALA’s efficacy in alleviating hepatic lipid accumulation.

### 3.2. ALA Mitigates HGHF-Induced Lipid Accumulation via TFEB in NCTC 1469 Cells

**Figure 2:**
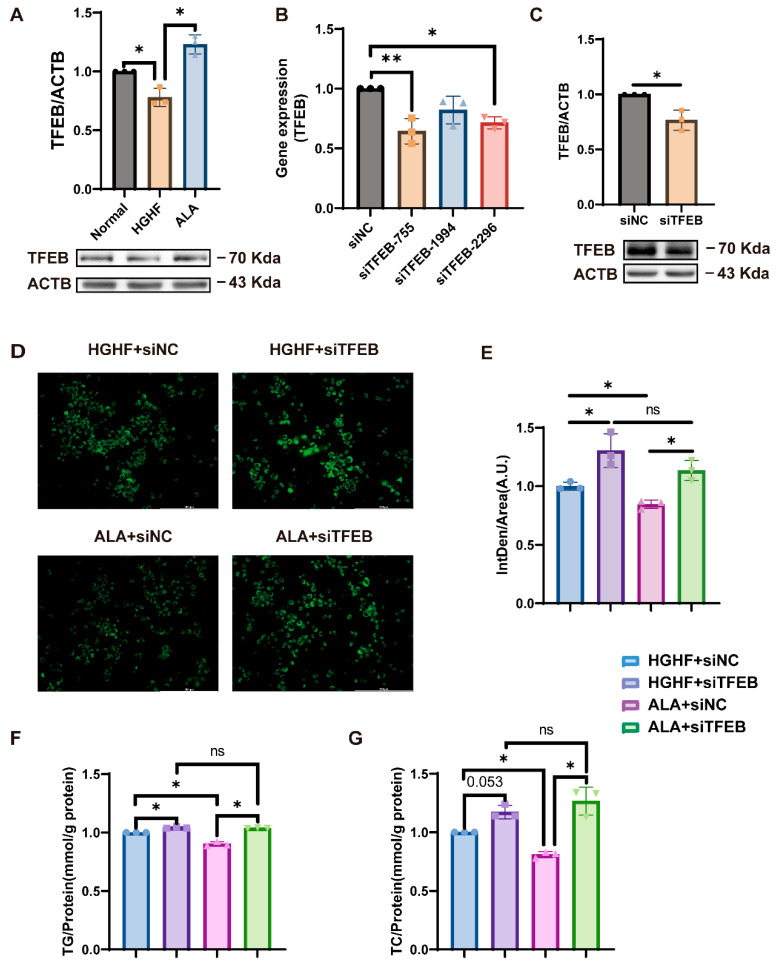
ALA mitigates HGHF-induced lipid accumulation via TFEB in cells. (**A**) ALA increased the HGHF-induced decrease in TFEB expression. (**B**) siRNA-TFEB-755 markedly reduced TFEB mRNA levels. (**C**) Protein expression of TFEB after transfection. (**D**) BODIPY 493/503 staining revealed enhanced lipid deposition in cells with TFEB knockdown under both HGHF and ALA conditions. (**E**) Quantification of BODIPY 493/503 fluorescence intensity is shown in Figure 2D. (**F**) TFEB knockdown led to elevated TG content in cells treated with HGHF and ALA. (**G**) TFEB knockdown led to elevated TC content in cells treated with HGHF and ALA. HGHF + si-NC, HGHF + siRNA-negative control; HGHF + si-TFEB, HGHF + siRNA-TFEB; ALA + si-TFEB, ALA + siRNA-TFEB. * *p* < 0.05, ** *p* < 0.01, ns *p* > 0.05.

TFEB has a pivotal function in lipid metabolism. In exploring the mechanism of ALA-alleviated lipid accumulation, we found that TFEB expression was significantly diminished in the HGHF group versus the normal group. However, this decrease was reversed following ALA treatment (Figure 2A).

We subsequently used siRNA to knockdown TFEB to further investigate its role in ALA’s alleviation of hepatocyte lipid accumulation. As shown in Figure 2B, transfection of NCTC 1469 cells with siRNA-TFEB-755, siRNA-TFEB-1994, or siRNA-TFEB-2296 markedly reduced TFEB expression, with greater knockdown efficiency in the siRNA-TFEB-755 group. Consequently, we selected siRNA-TFEB-755 (henceforth referred to as si-TFEB) for the next experiments. Western blotting confirmed that the level of TFEB decreased after treatment with si-TFEB (Figure 2C). As shown in Figure 2D–G, BODIPY 493/593 staining and TG and TC measurements showed that si-TFEB increased the HGHF-induced intracellular lipid accumulation, while ALA treatment reduced it. However, knocking down TFEB had no appreciable effect on lipid accumulation relative to the HGHF and ALA groups. This suggests that ALA downregulates HGHF-induced steatosis in hepatocytes by promoting TFEB expression.

### 3.3. ALA Promotes FA β-Oxidation via Upregulating TFEB Expression in HGHF-Induced NCTC 1469 Cells

**Figure 3:**
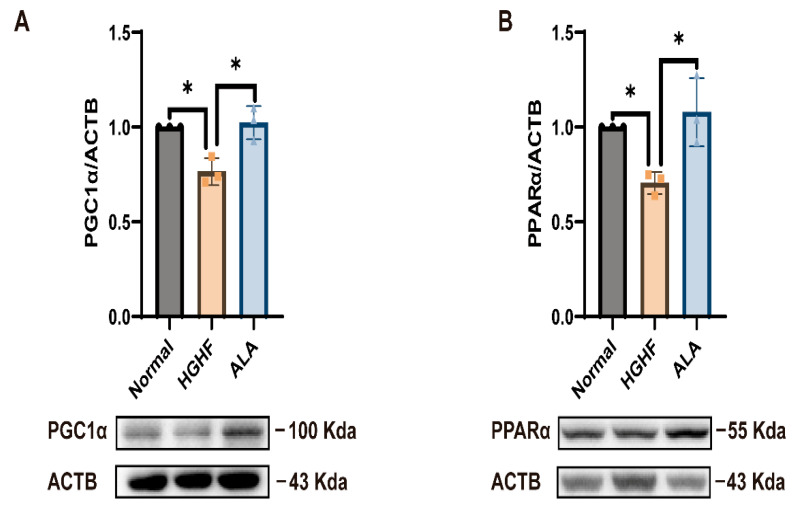

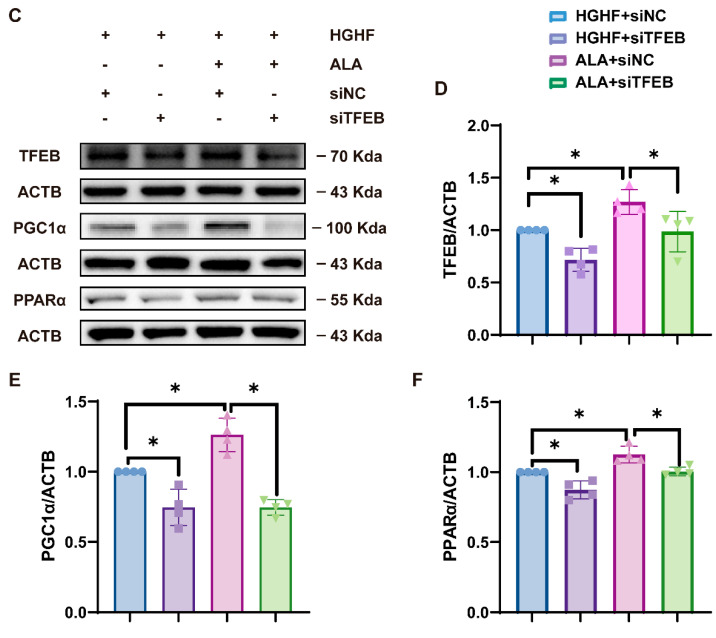
ALA promotes FA β-oxidation via upregulating TFEB expression in HGHF-induced cells. (**A**,**B**) ALA restored the expression of PPARα and PGC1α, previously reduced by HGHF treatment. (**C**–**F**) TFEB knockdown reduced TFEB, PPARα and PGC1α expression in cells treated with HGHF and ALA. * *p* < 0.05.

In order to examine the mechanism by which ALA inhibits lipid buildup in NCTC 1469 cells, we examined the changes in protein levels of β-oxidation-related proteins such as PPARα and PGC1α. PPARα and PGC1α levels were substantially diminished in the HGHF group versus the normal group, while ALA administration effectively restored their expression (Figure 3A,B).

Next, we investigated the changes in the levels of PPARα and PGC1α proteins after ALA treatment and TFEB knockdown to determine whether the regulation of β-oxidation by ALA is modulated by TFEB. Our results indicated that following TFEB knockdown in NCTC 1469 cells, TFEB and PPARα and PGC1α expression levels remained largely unchanged when comparing the HGHF to the ALA-treat group (Figure 3C–F). This finding suggests that ALA improves reduced β-oxidation in the HGHF group by the promotion of TFEB expression, thereby reducing lipid accumulation.

### 3.4. ALA Promotes CMA via Facilitating TFEB Expression in HGHF-Induced NCTC 1469 Cells

**Figure 4:**
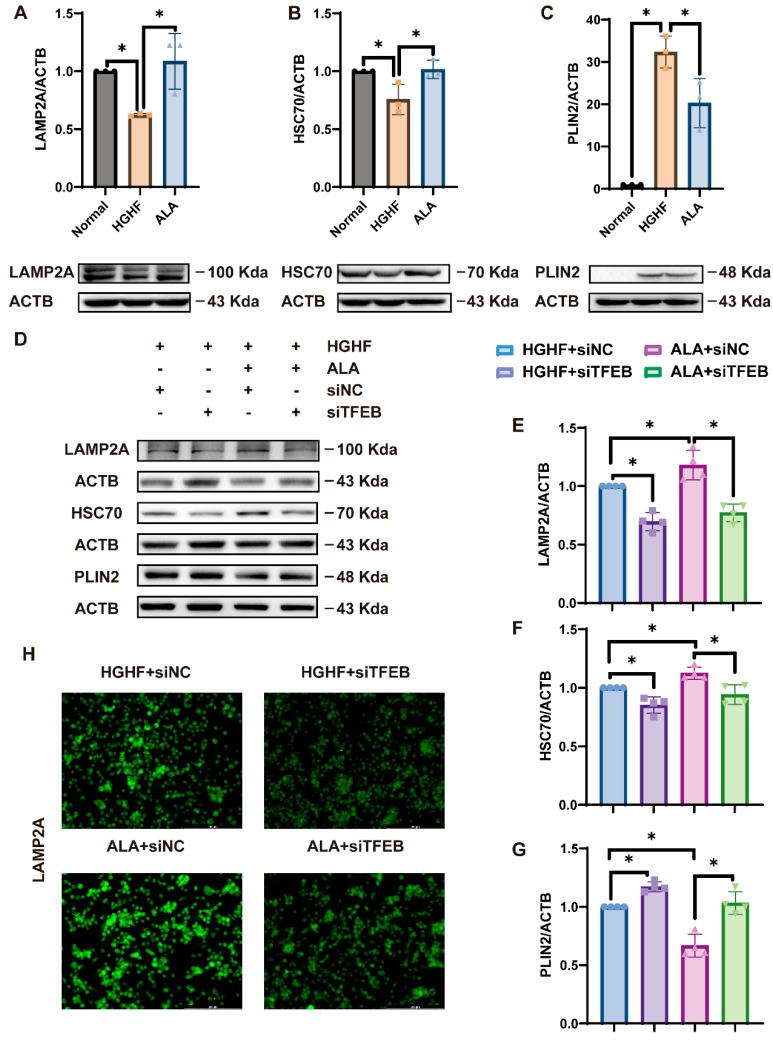
ALA promotes CMA via facilitating TFEB expression in HGHF-induced cells. (**A**–**C**) In NCTC 1469 cells, ALA increased the HGHF-induced decrease in LAMP2A and HSC70 the expression and, decreased the HGHF-induced increase in PLIN2 expression. (**D**–**G**) TFEB knockdown reduced LAMP2A and HSC70 levels while increasing PLIN2 expression in cells treated with HGHF and ALA. (**H**) Immunofluorescence analysis showed that ALA markedly upregulated LAMP2A expression, which was attenuated by TFEB knockdown. * *p* < 0.05.

In our study, we detected changes in CMA relative to the normal group: LAMP2A and HSC70 protein levels were significantly decreased in the HGHF group, while PLIN2 protein was elevated. These phenomena were reversed after ALA treatment (Figure 4A–C). Thus, we suggest that ALA can promote CMA expression.

The influence of ALA on regulating CMA-associated proteins was assessed in cells transfected with si-TFEB. Our results showed that ALA reversed the HGHF-induced decrease in LAMP2A and HSC70 and the HGHF-induced increase in PLIN2 expression; these effects were attenuated by the addition of si-TFEB (Figure 4D–G). Meanwhile, immunofluorescence detection, we concluded that ALA promoted the expression of LAMP2A by activating TFEB (Figure 4H). However, TFEB knockdown did not noticeably affect the levels of CMA-related proteins in either the HGHF or ALA groups. This finding suggests that ALA may mitigate the effects of HGHF on CMA by promoting TFEB and thus LAMP2A, which in turn reduces lipid accumulation.

### 3.5. ALA Promotes CMA via Facilitating NRF2 Expression in HGHF-Induced NCTC 1469 Cells

**Figure 5:**
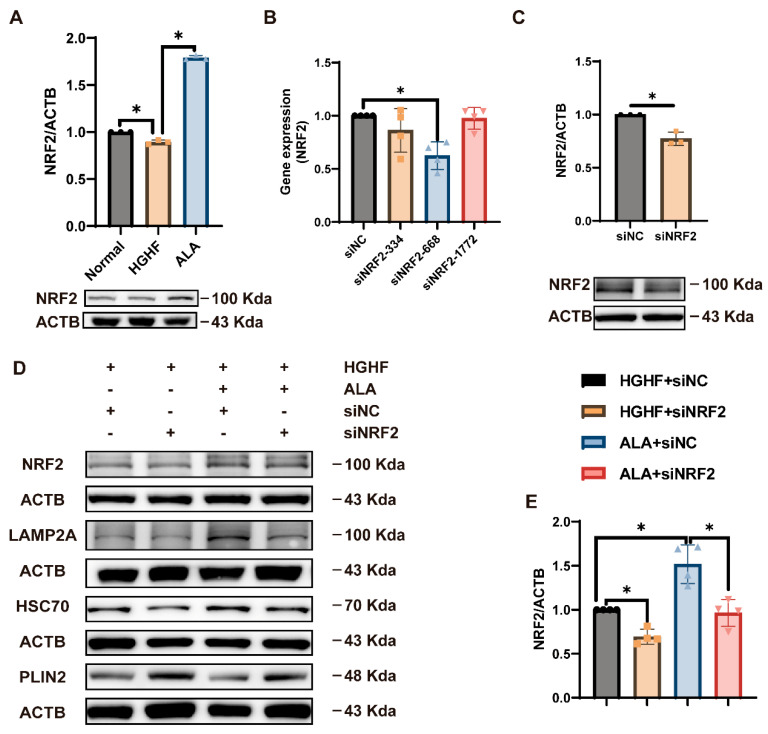

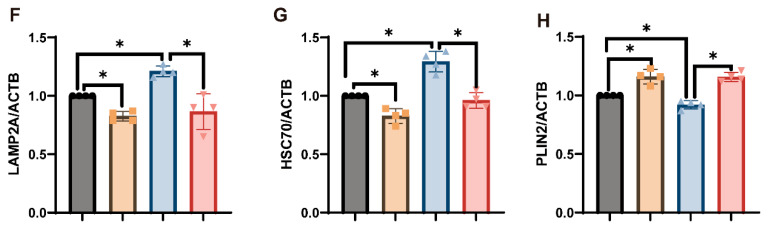
ALA promotes CMA via facilitating NRF2 expression in HGHF-induced cells. (**A**) In NCTC 1469 cells, ALA restored NRF2 expression that was has been by HGHF treatment. (**B**) siRNA-NRF2-668 markedly downregulated NRF2 mRNA expression. (**C**,**D**) Protein expression of NRF2 after transfection. (**D**–**H**) NRF2 knockdown reduced LAMP2A and HSC70 levels while elevating PLIN2 expression in cells treated with HGHF and ALA. * *p* < 0.05.

NRF2 expression was markedly lower in the HGHF group relative to the normal group, whereas ALA treatment restored its levels (Figure 5A). To further explore these factors’ role in CMA, cells were transfected with siRNA-NRF2-334, siRNA-NRF2-668, and siRNA-NRF2-1772. As a result, NRF2 expression was markedly suppressed, with the highest knockdown efficiency observed in the siRNA-NRF2-668 group. Thus, siRNA-NRF2-668 (hereafter referred to as si-NRF2) was chosen for subsequent experiments (Figure 5B). NRF2 protein expression was examined by Western blot analysis, which revealed a marked reduction in its protein level after si-NRF2 treatment (Figure 5C,D).

Then, we transfected NCTC 1469 cells with si-NRF2. Our results showed that ALA reversed the HGHF-induced decrease in LAMP2A and HSC70 and the HGHF-induced increase in PLIN2 expression; these effects of ALA were attenuated by the addition of si-NRF2. However, after silencing NRF2, the expression of CMA-related proteins showed no marked difference between the HGHF and ALA groups (Figure 5D–G). It can be concluded that NRF2 is involved in the promotion of CMA by ALA, thereby decreasing hepatocyte lipid accumulation.

### 3.6. ALA Alleviates Oxidative Stress in HGHF-Induced NCTC 1469 Cells

**Figure 6:**
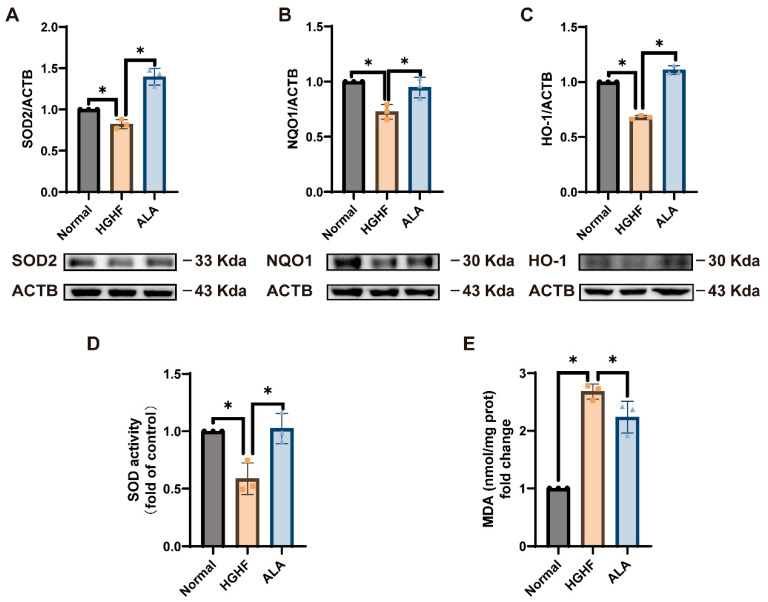

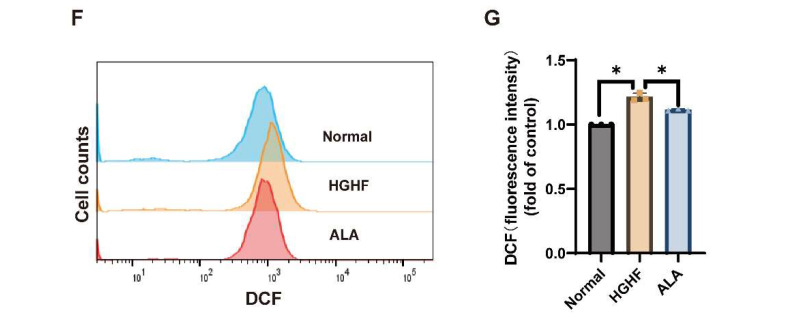
ALA alleviates oxidative stress in HGHF-induced cells. (**A**–**C**) In NCTC 1469 cells, ALA increased the HGHF-induced decrease in the expression of SOD2, NQO1, and HO-1. (**D**) SOD activity. (**E**) MDA level. (**F**,**G**) Representative peak plots showing intracellular ROS levels measured by flow cytometry using the DCFH-DA fluorescent probe. * *p* < 0.05.

To assess ALA’s impact on oxidative stress in HGHF-induced NCTC 1469 cells, we monitored oxidative stress-related proteins. Compared with the normal group, the HGHF group had substantially lower SOD2, NQO1, and HO-1 expression; these phenomena were markedly reversed by ALA (Figure 6A–C). As demonstrated in Figure 6D, SOD activity was significantly decreased in the HGHF group but was increased by ALA. Figure 6E shows that MDA levels were markedly elevated in the HGHF group, whereas they were considerably decreased in the ALA group. Flow cytometry revealed that the normal group had low basal ROS, while HGHF induction elevated intracellular ROS, an indicator of oxidative stress and inflammation. ALA treatment decreased this elevated ROS (Figure 6F,G). In conclusion, ALA mitigates HGHF-induced oxidative stress.

### 3.7. ALA Promotes TFEB, NRF2, and CMA in HGHF-Induced NCTC 1469 Cells via Activating AMPK (Thr172) Phosphorylation

**Figure 7:**
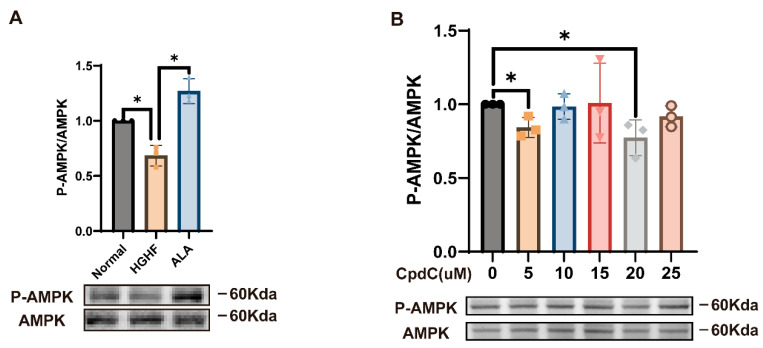

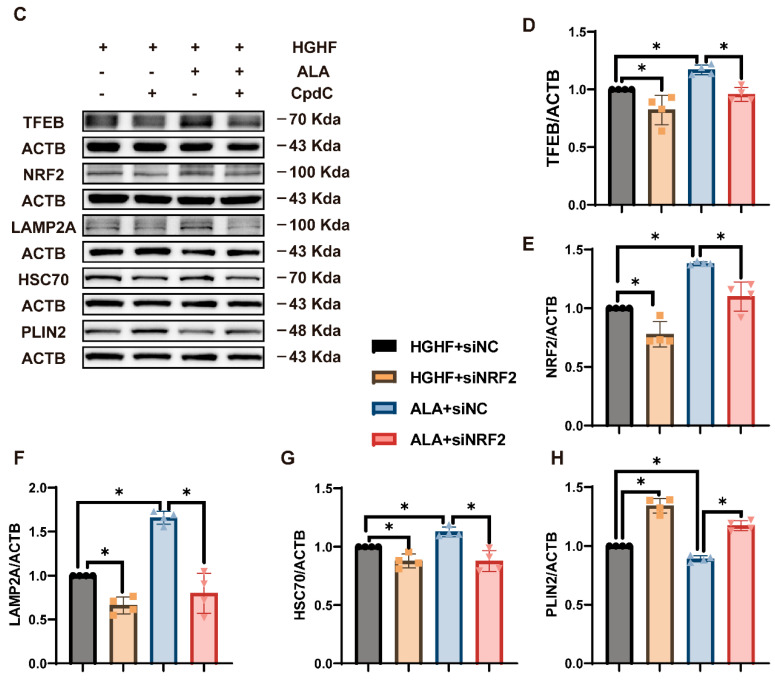
ALA promotes TFEB, NRF2, and CMA in HGHF-induced cells via activating AMPK (Thr172) phosphorylation. (**A**) ALA restored AMPK phosphorylation levels that had been reduced by HGHF treatment. (**B**) Impact of varying CpdC concentrations on AMPK phosphorylation in cells. (**C**–**H**) CpdC reversed the effect of ALA on induction in the HGHF group. * *p* < 0.05.

To further elucidate the mechanism by which ALA suppresses lipid accumulation and oxidative stress in HGHF-induced NCTC 1469 cells, we examined AMPK expression and its phosphorylated form (*p*-AMPK). Western blot analysis demonstrated a marked reduction in *p*-AMPK in the HGHF group relative to the normal group, whereas this decline was restored upon ALA administration (Figure 7A).

In addition, to determine whether AMPK mediates the regulatory effects of ALA on TFEB, the CMA-related proteins, and NRF2, we employed CpdC to decrease AMPK phosphorylation. Initially, we performed a Western blot assay to identify the optimal working concentration of CpdC. Based on the findings, 20 µM was chosen for subsequent experiments (Figure 7B). Then, NCTC 1469 cells were pretreated with CpdC for 1 h to inhibit AMPK activation, followed by treatment with HGHF and ALA for an additional 24 h. We found that ALA promoted the expression of TFEB, LAMP2A, HSC70, and NRF2 while inhibiting PLIN2 expression, whereas these effects were abolished by CpdC treatment. In contrast, after addition of CpdC, no marked difference in the expression of TFEB and NRF2 and CMA-related proteins was observed between the HGHF group and the ALA group. These results suggest that ALA promotes TFEB, NRF2, LAMP2A, and HSC70 expression and inhibits PLIN2 expression by the activation of the AMPK pathway (Figure 7C–H). Thus, ALA alleviates lipid accumulation and oxidative stress by activating AMPK.

### 3.8. ALA Raises CMA Activity via Activating the AMPK-TFEB/NRF2 Axis in NCTC 1469 Cells

**Figure 8:**
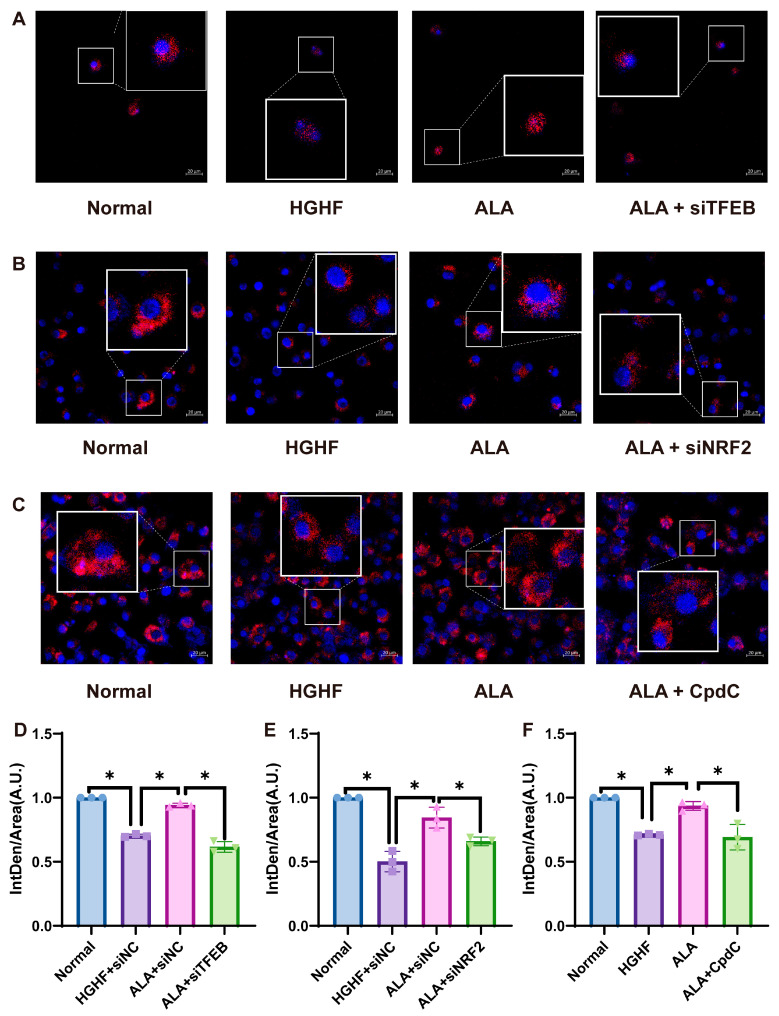
ALA raises CMA activity via activating the AMPK-TFEB/NRF2 axis in cells. (**A**–**F**) Representative images showing mCherry fluorescent puncta for each treatment group (scale bar = 20 μm). * *p* < 0.05.

To further observe the changes in CMA, we assessed the effects of ALA, siTFEB, siNRF2, and Cpdc on CMA activity using PA-mCherry-KFERQ. Quantitative assessment demonstrated a notable loss of fluorescent puncta in the HGHF group compared to the control, which was largely restored following ALA administration. Additionally, the number of fluorescence points decreased following combined treatment with ALA and siTFEB, or ALA and siNRF2, compared to treatment with ALA alone (Figure 8A–C). The data support the conclusion that ALA counteracts the down-regulation of CMA activity induced by HGHF via TFEB and NRF2. Furthermore, the number of fluorescence points was significantly reduced following co-treatment with ALA and CpdC, which further demonstrates that ALA can activate CMA via the AMPK-TFEB/NRF2 axis.

### 3.9. ALA Attenuated Liver Damage, Hepatic Steatosis, and Elevated Serum Lipid Levels in HFD-Fed Mice

In order to characterize the functional involvement of ALA in NAFLD, NAFLD was initially induced in mice using an HFD. Five HFD- mice were then randomly selected to be treated with oral ALA.

**Figure 9:**
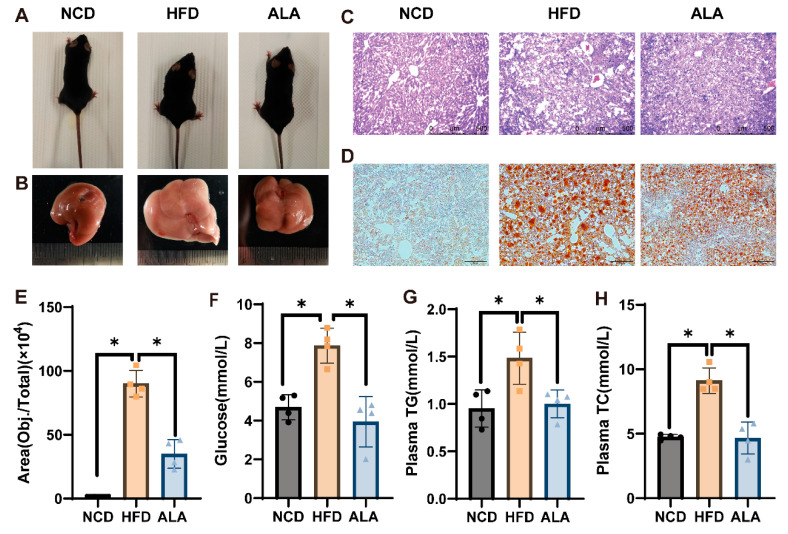

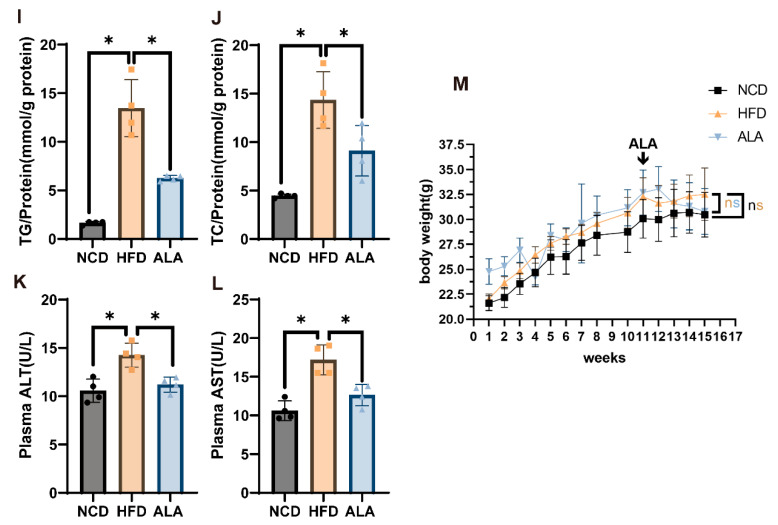
ALA attenuated liver damage, hepatic steatosis, and elevated serum lipid levels in mice fed an HFD. (**A**) Mouse status. (**B**) Mouse livers. (**C**) Representative H&E-stained images of liver tissue. (100×) (*n* = 4). (**D**) Representative Oil Red O-stained images of liver tissue (*n* = 4). (**E**) Quantification of Oil Red O staining shown in Figure 8D. ALA reduced the levels of plasma glucose (**F**) (*n* = 4), plasma TG (**G**) (*n* = 4), plasma TC (**H**) (*n* = 4), liver TG (**I**) (*n* = 4), liver TC (**J**) (*n* = 4), plasma ALT (**K**) (*n* = 4), and plasma AST (**L**) (*n* = 4) in the HFD-induced group. (**M**) Weekly body weight changes in the mice. * *p* < 0.05, ns *p* > 0.05.

As shown in Figure 9A, the HFD mice had rough and greasy fur and were fat, while mice in the ALA group had cleaner fur and a thinner body type. Figure 9B illustrates that HFD feeding resulted in livers with a yellowish coloration, decreased elasticity, and a granular appearance, distinct from the healthy livers of the NCD group. The morphological changes in the livers of mice were examined using HE staining and Oil Red O staining. As shown in Figure 9C–E, the HFD group exhibited significant hepatocyte ballooning and lipid droplet accumulation compared with the NCD group. After ALA treatment, the ballooning was diminished and the lipid accumulation was reduced. Then, we detected the contents of blood glucose, blood lipid, and liver lipid in the mice. The results showed that HFD feeding led to marked elevations in the contents of glucose, liver TG, liver TC, plasma TG, and plasma TC in relation to the levels in the NCD group. Moreover, the aforementioned increases were markedly decreased in the ALA group relative to those observed in the HFD group (Figure 9F–J). In addition, we monitored the levels of the liver injury markers plasma ALT and AST. They rose notably in the HFD group, whereas in the NCD group, they remained unchanged. Similarly, ALA administration markedly reduced the above-mentioned changes (Figure 9K–M). Figure 9M illustrates the progression of weekly body weight in the mice, with no significant differences observed. However, the mice gained weight after one week on the HFD. After 11 weeks on the HFD, those administered oral ALA for 2 weeks showed a downward trend in body weight. These findings suggest that ALA ameliorates HFD-induced NAFLD through attenuating hepatic lipid accumulation, liver injury, and abnormal glucose elevation.

### 3.10. ALA Improves AMPK-TFEB/NRF2 Axis-Mediated CMA, β-Oxidation, and Antioxidant Levels in HFD Mice

**Figure 10:**
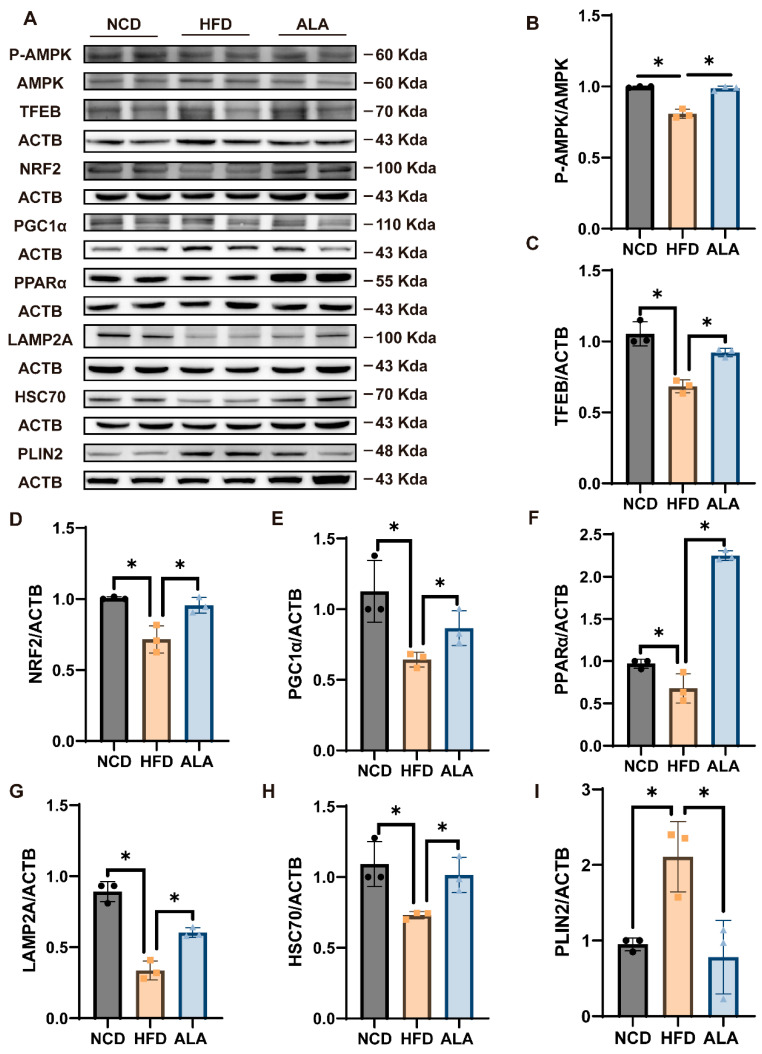
ALA improves AMPK-TFEB/NRF2 axis-mediated CMA, β-oxidation, and antioxidant levels in HFD mice. (**A**) Effects of HFD and ALA treatment on relevant proteins in AMPK-TFEB/NRF2. Quantitative analysis of *p*-AMPK (**B**), TFEB, NRF2 (**C**,**D**) PGC1α, PPARα (**E**,**F**) and LAMP2A, HSC70, and PLIN2 (**G**–**I**). Quantitative analysis results. * *p* < 0.05.

To further confirm the effect of ALA in alleviating NAFLD in mice, the expression of AMPK, TFEB, NRF2, PGC1α, PPARα, LAMP2A, HSC70, and PLIN2 was detected using Western blotting in liver tissues. Figure 10 shows that HFD mice exhibited substantial reduction in *p*-AMPK, TFEB, LAMP2A, HSC70, PGC1α, PPARα and NRF2, while PLIN2 levels were increased compared to the NCD group. Subsequently, ALA treatment reversed these effects in the HFD-induced mice. These data indicate that ALA exerts protective effects against NAFLD through stimulation of the AMPK-TFEB/NRF2 pathway.

## 4. Discussion

**Figure 11:**
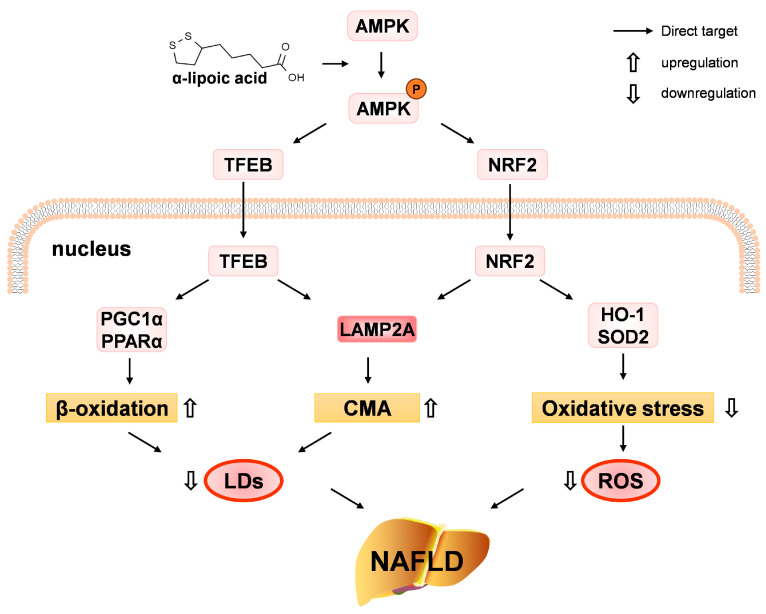
A schematic diagram showing that ALA alleviates NAFLD by modulating AMPK-TFEB/NRF2 mediated activation of β-oxidation and CMA and oxidative stress suppression.

Our investigation showed that ALA, by triggering the AMPK-TFEB/NRF2 axis, ameliorated impaired CMA, β-oxidation, and antioxidant levels, thereby counteracting HGHF-induced lipid accumulation and oxidative stress in hepatocytes (NCTC 1469 cells). Further studies demonstrated that ALA exerted a protective effect against hepatic steatosis and liver injury in HFD-induced NAFLD mice (Figure 11).

The current range of approved pharmacological treatments is largely limited to advanced stages of NAFLD/NASH. This highlights the need for effective intervention strategies that target the early stages of the disease.

ALA has been extensively studied as an antioxidant. ALA has been shown to attenuate steatohepatitis induced by a methionine-deficient and choline-deficient diet in mice [38]. Over the past year, the medicinal value of ALA has also been demonstrated in cases of rat liver injury and pulmonary fibrosis [39,40]. An excessive intake of sugar and fat is known to elevate the risk of developing NAFLD. To investigate the pathogenesis of NAFLD and the effects of ALA, we successfully established a hepatocytic steatosis model in NCTC 1469 cells induced by OA/PA combined with glucose (HGHF) in vitro, characterized by significantly increased cell lipid accumulation after 24 h. Moreover, we found that ALA inhibited lipid accumulation induced by the HGHF treatment in NCTC 1469 cells (Figure 1C–J).

Excessive hepatic lipid accumulation is a key driver of NAFLD progression, making the regulation of lipid homeostasis a central therapeutic target [41]. Growing evidence indicates that TFEB participates in multiple metabolic processes, including lipid metabolism [42]. Previous studies have shown that TFEB is impaired in alcohol-associated liver disease (ALD) [43] and that enhancing TFEB expression alleviates hepatic steatosis [44]. Consistent with these findings, our study showed that HGHF exposure reduced TFEB in hepatocytes, whereas ALA restored its expression (Figure 2A). Furthermore, defective TFEB promotes lipid accumulation in NCTC 1469 cells (Figure 2D–G), suggesting that TFEB plays a functional role in hepatic lipid homeostasis.

Mechanistically, TFEB has been shown to regulate lipid catabolism through a transcriptional network involving PGC1α and PPARα, key regulators of β-oxidation [10]. Impaired β-oxidation is a well-established contributor to hepatic lipid accumulation [45], as evidenced by severe hepatic steatosis in PARα-deficient mice [46]. In line with these studies, we found that TFEB knockdown reduced the expression of PGC1α and PPARα, the β-oxidation-related genes (Figure 3). Collectively, our results support previous studies and demonstrate that ALA alleviates hepatic lipid accumulation by upregulating the TFEB–β-oxidation axis.

Some studies have shown dysfunctional CMA function during the progression of hepatic steatosis in both NAFLD patients and HFD-induced animal models [14]. Hepatocytes with CMA impairment due to LAMP2A deletion exhibited enhanced LD accumulation and increased serum TG levels in HFD-fed mice [47]. In line with this concept, our studies suggest that ALA can enhance HGHF-induced reduced expression of LAMP2A and HSC70 and decrease HGHF-induced increased expression of PLIN2 (Figure 4A–C). These results also showed that ALA improved NAFLD by increasing CMA. To further elucidate its regulatory mechanisms, we examined transcription factors previously implicated in CMA modulation. In this study, the HGHF-induced decreased expression of LAMP2A and HSC70 was lower with TFEB knockdown, while the HGHF-induced increased expression of PLIN2 was higher. However, the effect of si-TFEB was attenuated by treatment with ALA (Figure 4D–G). The trend of CMA-related proteins after the addition of si-NRF2 was consistent with that of TFEB knockdown (Figure 5E–H). Furthermore, by fusing NCTC 1469 cells with PA-mCherry-KFERQ, we directly demonstrated that ALA increases CMA activity via TFEB/NRF2 (Figure 8A–C). Taken together, these observations indicate that ALA mitigates NAFLD by potentiating TFEB/NRF2-mediated CMA, consequently augmenting lipid droplet catabolism.

Furthermore, according to the “multiple hit” hypothesis, oxidative stress is considered a key driver of hepatic injury and the advancement of NAFLD. Oxidative stress arises when reactive oxygen species (ROS) generation overwhelms the antioxidant defense mechanisms, causing a shift that favors the buildup of ROS [48]. NRF2 is an inducible transcription factor, that accumulates in the nucleus under oxidative stress conditions, where it binds to antioxidant response genes HO-1and NQO1 regulating multiple cellular antioxidant systems [49]. Our findings observed that ALA restored the expression of antioxidative stress proteins—NRF2, SOD2, HO-1, and NQO-1—downregulated by HGHF in hepatocytes (Figure 5A and Figure 6A–C). Consistently, ALA enhanced SOD activity, reduced MDA content, and attenuated ROS overproduction induced by HGHF (Figure 6D–G). In conclusion, the present results demonstrate that ALA provides protection against HGHF-induced NAFLD by reducing oxidative stress via increasing NRF2/HO-1/NQO1/SOD.

Accumulating evidence suggests that AMPK is a critical signaling target controlling the pathways of hepatic lipid metabolism, and its activation has been shown to alleviate NAFLD/NASH through pathways such as TFEB and NRF2 [23,50]. Our outcomes showed that AMPK phosphorylation was inhibited by HGHF and that ALA promoted this process. The AMPK inhibitor Cpd C reduced ALA-induced activation of TFEB and NRF2 (Figure 7C–E). Consequently, these findings collectively indicate that ALA mitigates NAFLD through the AMPK-TFEB/NRF2 core pathway. Although Cpd C is widely used to inhibit AMPK activity, due to its limited specificity and potential off-target effects, its specific impact should be interpreted in conjunction with our overall findings and additional experimental results.

Furthermore, NAFLD mouse models were constructed following a previously reported protocol [51], after which ALA treatment was administered. These results suggested that HFD induced liver injury and lipid accumulation in mice, which could be relieved by ALA (Figure 9C–E,K,L). Other results revealed that ALA lowered hepatic and serum levels of TG and TC, in comparison with the HFD group (Figure 9G–J). Subsequent analysis of proteins associated with AMPK revealed increased levels of *p*-AMPK, TFEB, NRF2, PPARα, PGC1α, LAMP2A, and HSC70 and decreased expression of PLIN2 (Figure 10). In conclusion, ALA alleviated NAFLD via activation of AMPK-TFEB/NRF2-mediated β-oxidation, CMA, and antioxidant pathways.

Longhitano et al. demonstrated that ALA alleviates NAFLD via regulating mitochondrial function, lipid metabolism, and oxidative stress in a cellular model [34,35,36]. Yang et al. showed that ALA attenuated insulin sensitivity and improved glucose homeostasis in HFD-fed mice [52]. The present study’s result aligns with prior research, indicating that ALA is an effective treatment for NAFLD. More importantly, we have demonstrated a novel mechanism through which ALA treats NAFLD in both cell and animal models. Our study revealed that ALA enhances the AMPK-TFEB pathway to promote fatty acid β-oxidation, upregulates the AMPK-NRF2 axis to alleviate oxidative stress, and activates CMA through the AMPK-NRF2/TFEB axis, thereby alleviating NAFLD. In summary, our research validates the therapeutic efficacy of ALA against NAFLD via the AMPK-TFEB/NRF2 in both in vivo and in vitro models, thereby providing novel therapeutic targets for this condition. The in vivo dose of ALA used in this study was selected to demonstrate efficacy in mice. Further clinical investigation is necessary to establish translationally relevant dosing in humans.

NAFLD has emerged as a major cause of hepatic dysfunction worldwide, though its precise pathophysiological mechanisms remain incompletely understood. While Rezdiffra was approved in 2024 for NASH with-moderate-to advanced liver fibrosis, no effective treatment exists for early-stage NAFL as of yet, making early diagnosis and management urgent research priorities. Ma et al. demonstrated significantly reduced CMA activity in non-fibrotic NAFLD mice [16]. Emerging evidence indicates that impaired hepatic lipophagy drives pathological progression throughout NAFLD [53,54]. The NAFLD model induced by a high-fat diet used in this study reflects key metabolic features of human NAFLD but only represents part of the disease spectrum. Therefore, the present findings are most relevant to early-stage NAFLD. To better define the contribution of ALA to NAFLD, further validation in alternative induction models is warranted.

## 5. Conclusions

In summary, our findings indicate that ALA protects against NAFLD by activating the AMPK-TFEB/NRF2 signaling pathway, which enhances β-oxidation and CMA and decreases oxidative stress. These results highlight the therapeutic potential of ALA and support its further development as a promising strategy for the prevention and management of NAFLD.

## Figures and Tables

**Table 1:** Primers for RT-qPCR.

Gene Symbol	NCBI Reference Sequence	Forward Primer (5′–3′)	Reverse Primer (5′–3′)
β-actin	NM_007393.5	GATCTGGCACCACACCTTCT	GGGGTGTTGAAGGTCTCAAA
TFEB	NM_011549.3	CCAGAAGCGAGAGCTCACAGAT	TGTGATTGTCTTTCTTCTGCCG
NRF2	NM_010902.5	CTCAGCATGATGGACTTGGA	TCTTGCCTCCAAAGGATGTC

## Data Availability

The original contributions presented in this study are included in the article/Supplementary Materials. Further inquiries can be directed to the corresponding author.

## Supplementary Materials

The following supporting information can be downloaded at: https://www.mdpi.com/article/10.3390/nu18030402/s1.

## Abbreviations

ALA	α-lipoic acid
AMPK	AMP-activated protein kinase
CMA	chaperone-mediated autophagy
CMC-Na	carboxymethyl cellulose solution
CpdC	dorsomorphin, Compound C
HFD	high-fat diet
HGHF	high glucose and high fat
HO-1	heme oxygenase-1
HSC70	heat shock cognate 71 Kda protein
LAMP2A	lysosomal-associated membrane protein 2
LDs	lipid droplets
NAFLD	non-alcoholic fatty liver disease
NCD	normal diet
NRF2	nuclear factor-erythroid 2-p45 derived factor 2
NQO-1	NAD(P)H: quinone oxidoreductase 1
OA	oleic acid
PA	palmitic acid
PGC1α	PPAR-γ co-activator-1 α
PLIN2	perilipin 2
PPARα	peroxisome proliferator-activated receptor α
ROS	reactive oxygen species
TFEB	transcription factor EB
VLDL	very-low-density lipoprotein
