# Supplementary material for: α-Lipoic Acid Alleviates Non-Alcoholic Fatty Liver Disease by Elevating Chaperone-Mediated Autophagy and Increasing β-Oxidation via AMPK-TFEB Axis
Source: Nutrients. 2026 Jan 26;18(3):402. doi: 10.3390/nu18030402 (PMC12899322; doi:10.3390/nu18030402)
Supplement: Supplementary file 1 [file nutrients-18-00402-s001.zip › Supplementary Information.pdf]

Supplemental Information

α-lipoic acid alleviates non-alcoholic fatty liver disease by elevating chaperone-mediated autophagy and increasing β-oxidation via AMPK-TFEB axis

Keting Dong[1], Miao Zhang[1], Jiaojiao Xu[1], Xue Bai[1], Jianhong Yang[1*]

[1] Medical School, University of Chinese Academy of Sciences, Beijing 101400, China

dongketing21@mails.ucas.ac.cn (K.D.); zhangmiao21@mails.ucas.ac.cn (M.Z.); 15733156243@163.com (J.X.); paihsue@foxmail.com (X.B.)

* Correspondence: yangjh@ucas.ac.cn (J.Y.)

Supplemental Figure

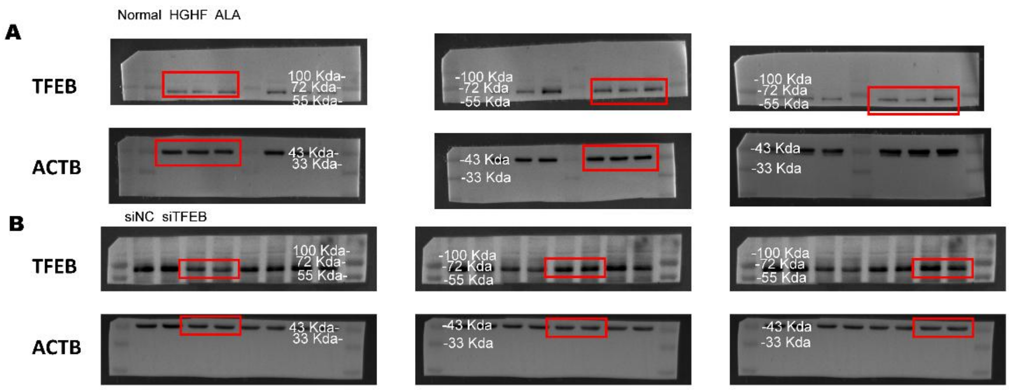

Figure S 1 Western blot full size image of NCTC 1469 cells. Figure A shows the NCTC 1469 cells were grouped into Normal, HGHF and ALA. Full-size western blot images of TFEB were used to evaluate the effect of ALA. Figure B shows full-size western blot images of TFEB after addition of si-TFEB in NCTC 1469 cells. The red box of a full-size western blot image is the area for grouping calculations.

Supplemental Information

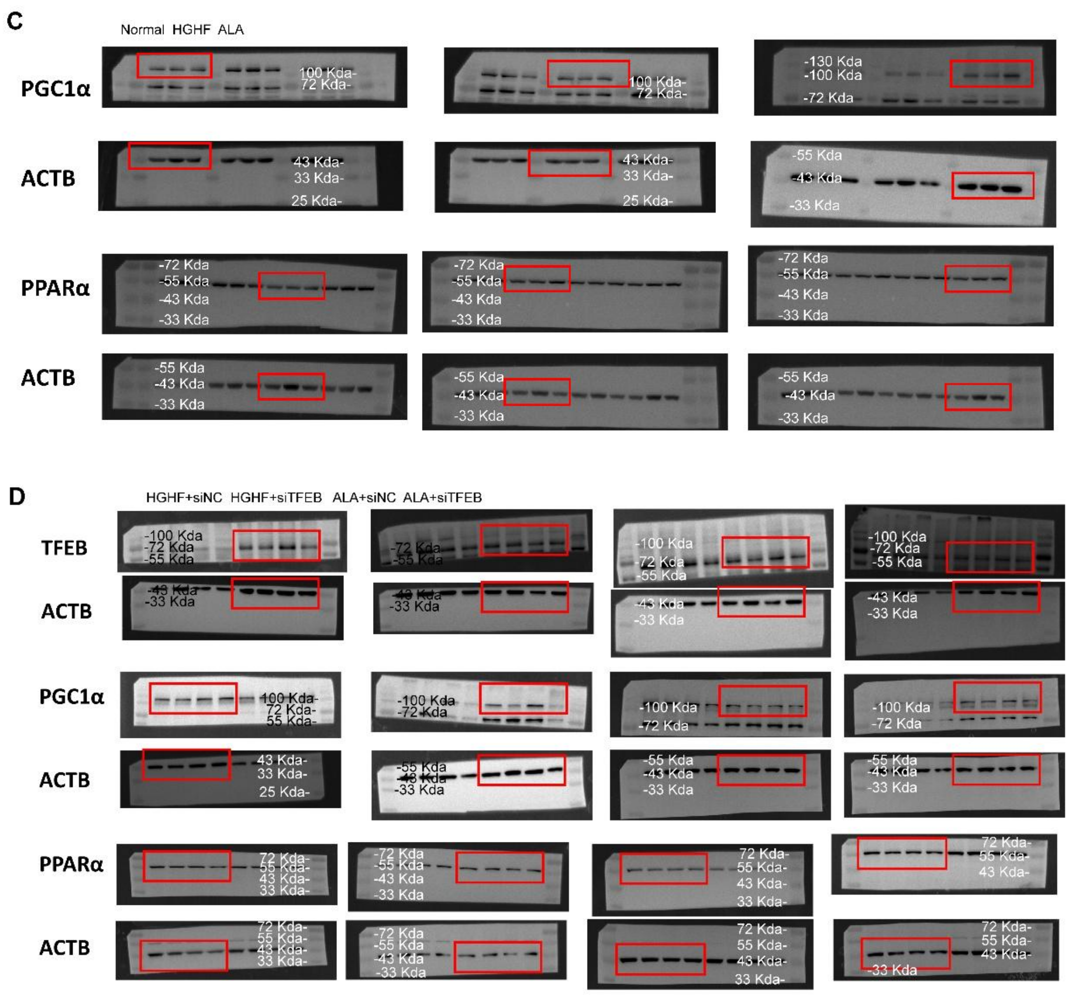

Figure S 2 Western blot full size image of NCTC 1469 cells. Figure C shows the NCTC 1469 cells were grouped into Normal, HGHF and ALA. Full-size western blot images of PGC1α and PPARα were used to evaluate ALA increased β-oxidation of HGHF-induced NCTC 1469 cells. Figure D shows full-size western blot images of TFEB and PGC1α and PPARα after addition of si-TFEB. The red box of a full-size western blot image is the area for grouping calculations.

Supplemental Information

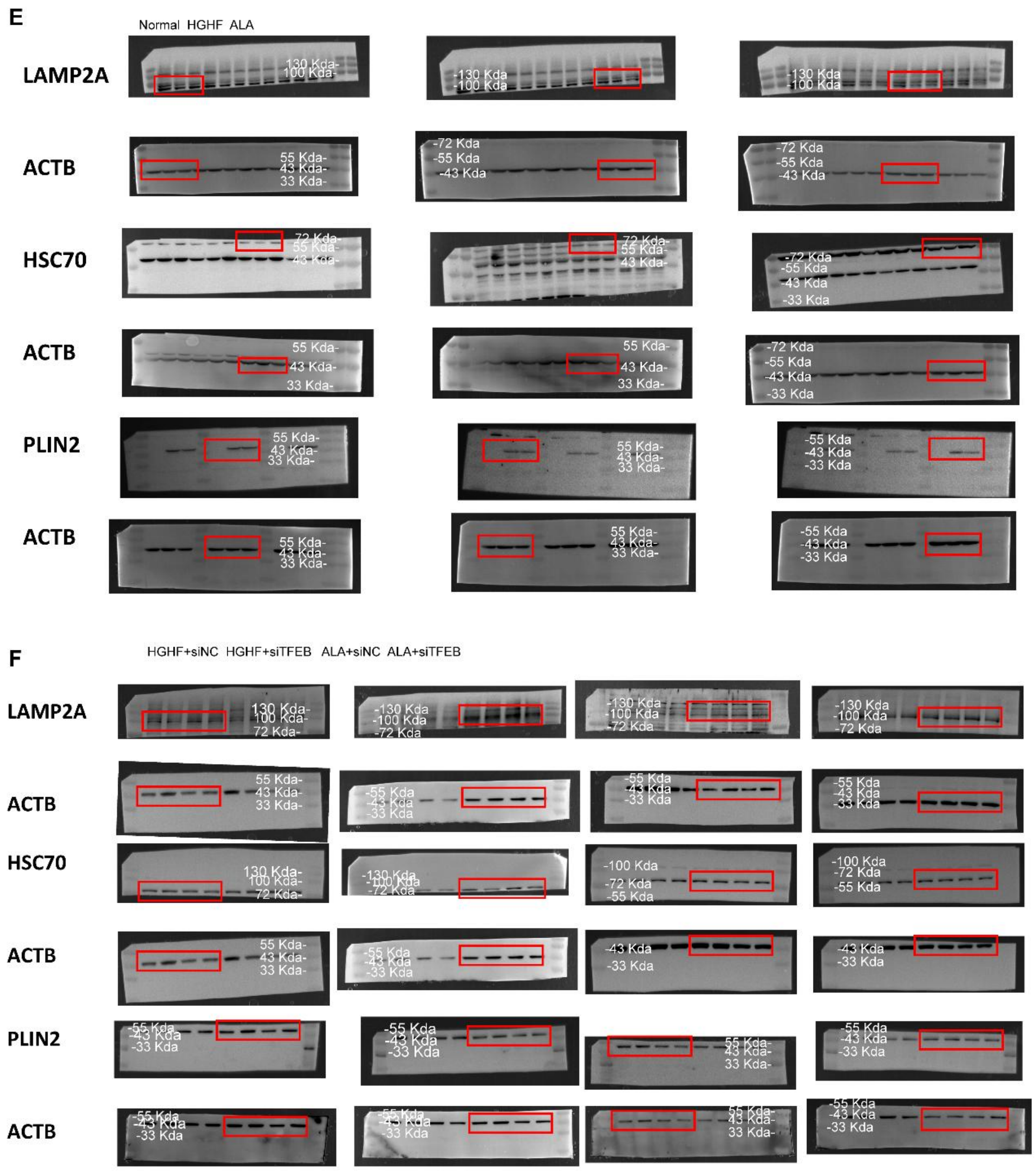

Figure S 3 Western blot full size image of NCTC 1469 cells. Figure E shows the NCTC 1469 cells were grouped into Normal, HGHF and ALA. Full-size western blot images of LAMP2A and HSC70 and PLIN2 were used to evaluate ALA increased CMA of HGHF-induced NCTC 1469 cells. Figure F shows full-size western blot images of LAMP2A and HSC70 and PLIN2 after addition of si-TFEB. The red box of a full-size western blot image is the area for grouping calculations.

Supplemental Information

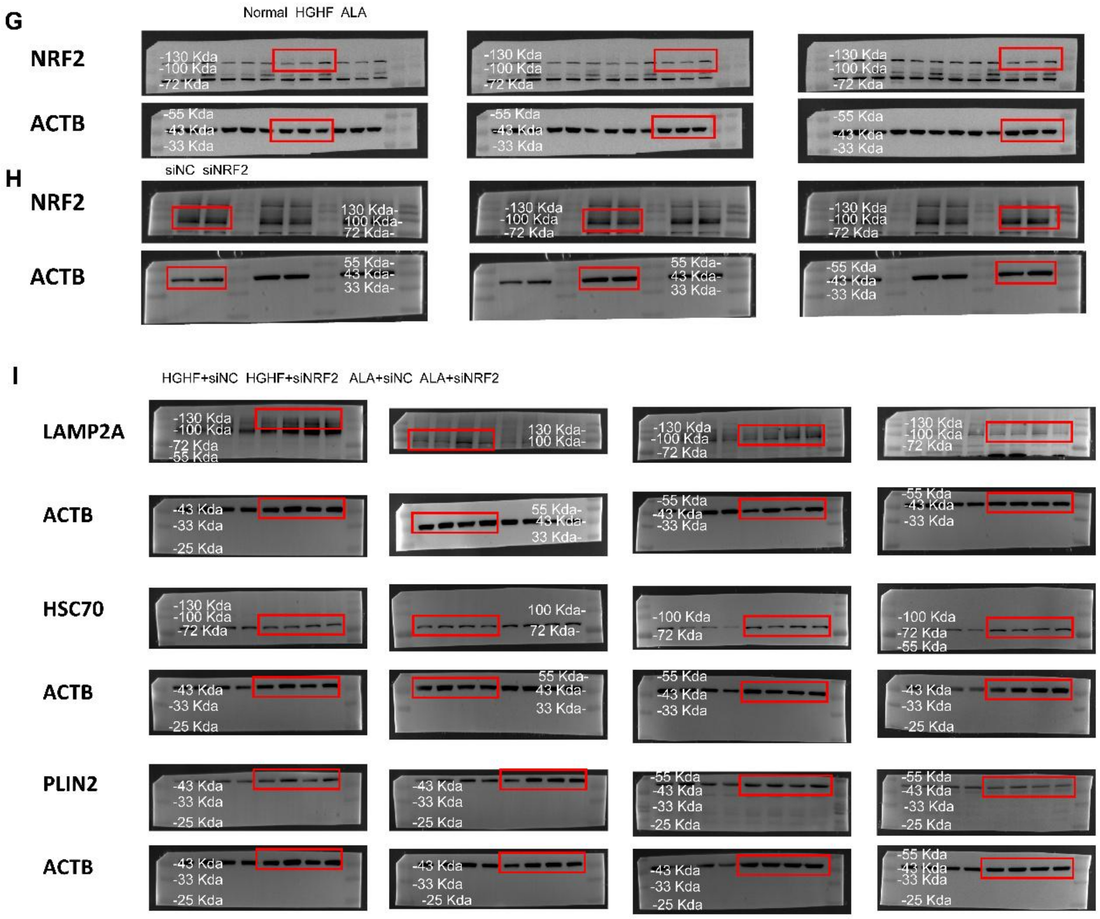

Figure S 4 Western blot full size image of NCTC 1469 cells. Figure G shows the NCTC 1469 cells were grouped into Normal, HGHF and ALA. Full-size western blot images of NRF2 were used to evaluate ALA increased antioxidant of HGHF-induced NCTC 1469 cells. Figure H shows full-size western blot images of NRF2 after addition of si-NRF2 in NCTC 1469 cells. Figure I shows full-size western blot images of LAMP2A and HSC70 and PLIN2 after addition of si-NRF2. The red box of a full-size western blot image is the area for grouping calculations.

Supplemental Information

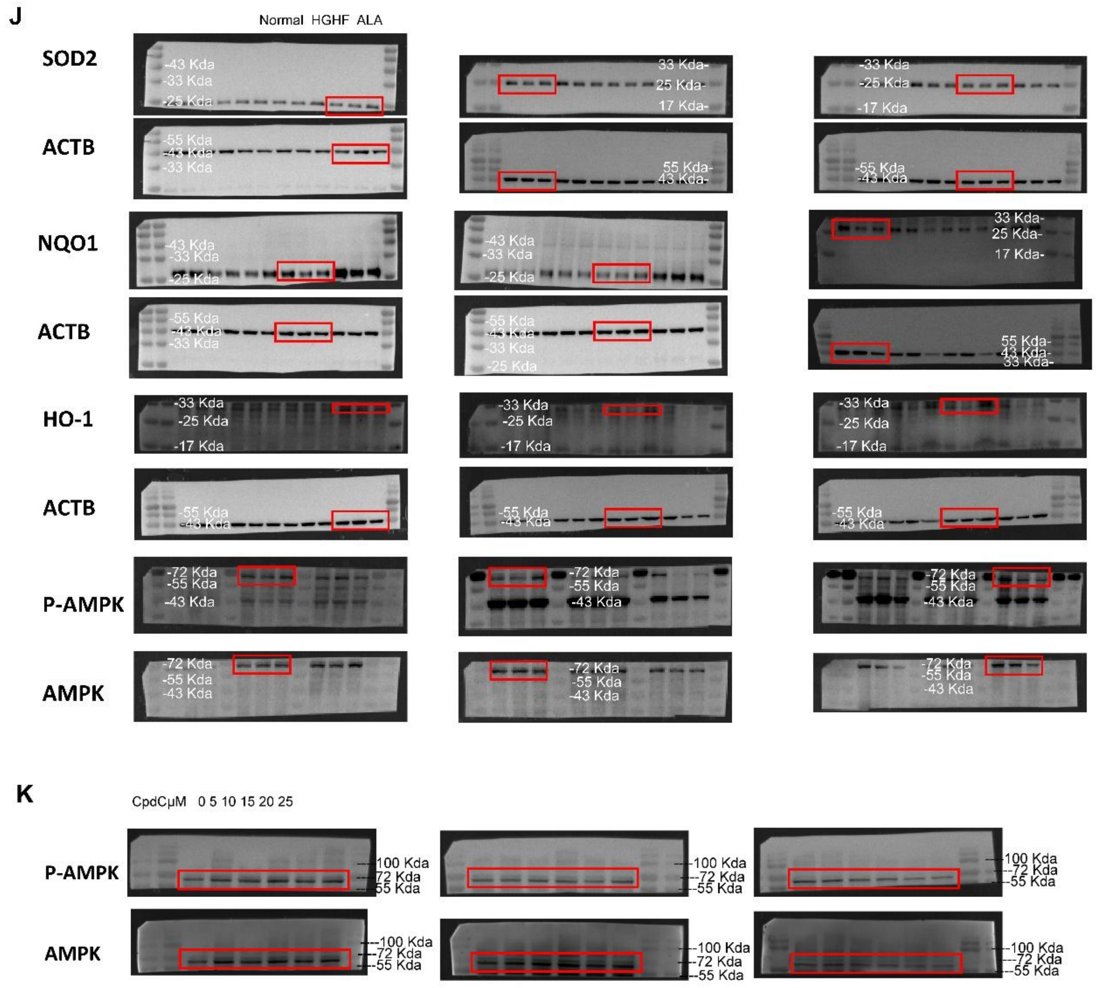

Figure S 5 Western blot full size image of NCTC 1469 cells. Figure J shows the NCTC 1469 cells were grouped into Normal, HGHF and ALA. Figure J is full-size western blot images of SOD2, NQO1, HO-1, p-AMPK and AMPK in NCTC 1469 cells.

Figure K shows full-size western blot images of p-AMPK and AMPK after addition of CpdC in NCTC 1469 cells. The red box of a full-size western blot image is the area for grouping calculations.

Supplemental Information

L

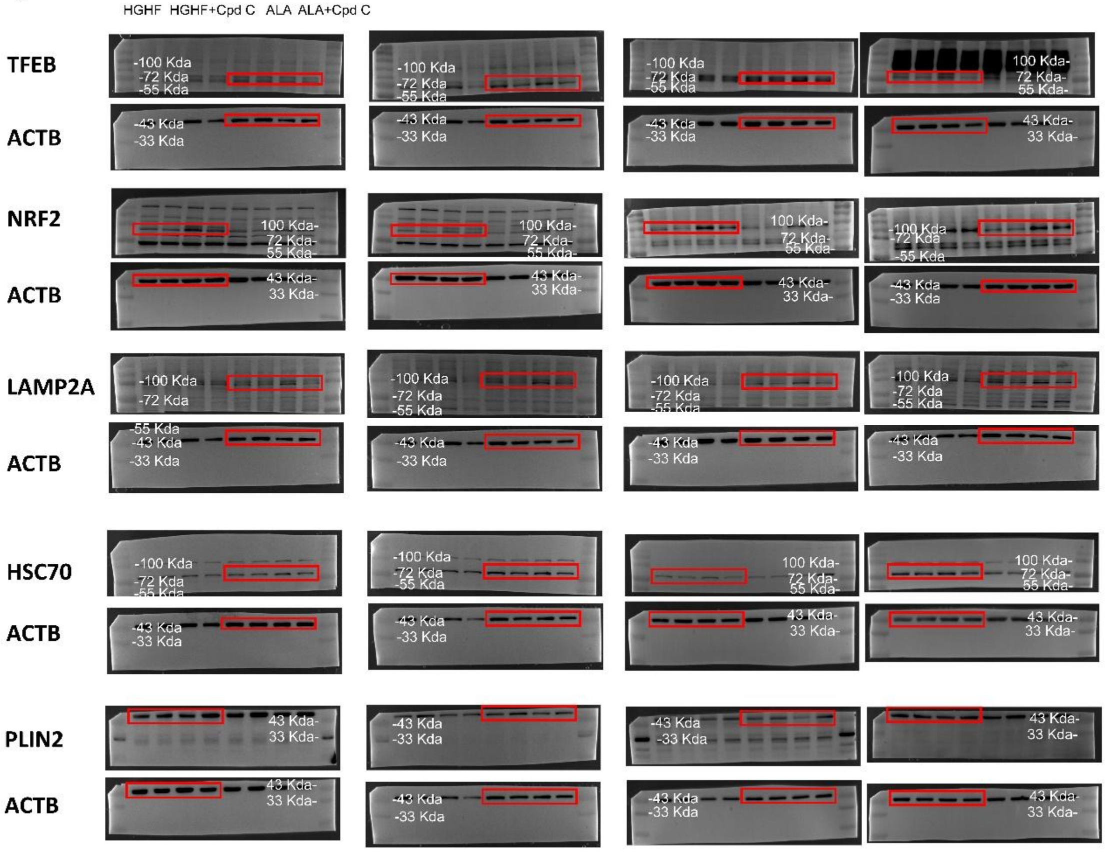

Figure S 6 Western blot full size image of NCTC 1469 cells. Figure L shows full-size western blot images of TFEB, NRF2, LAMP2A, HSC70 and PLIN2 in NCTC 1469 cells after the addition of CpdC. The red box of a full-size western blot image is the area for grouping calculations.

Supplemental Information

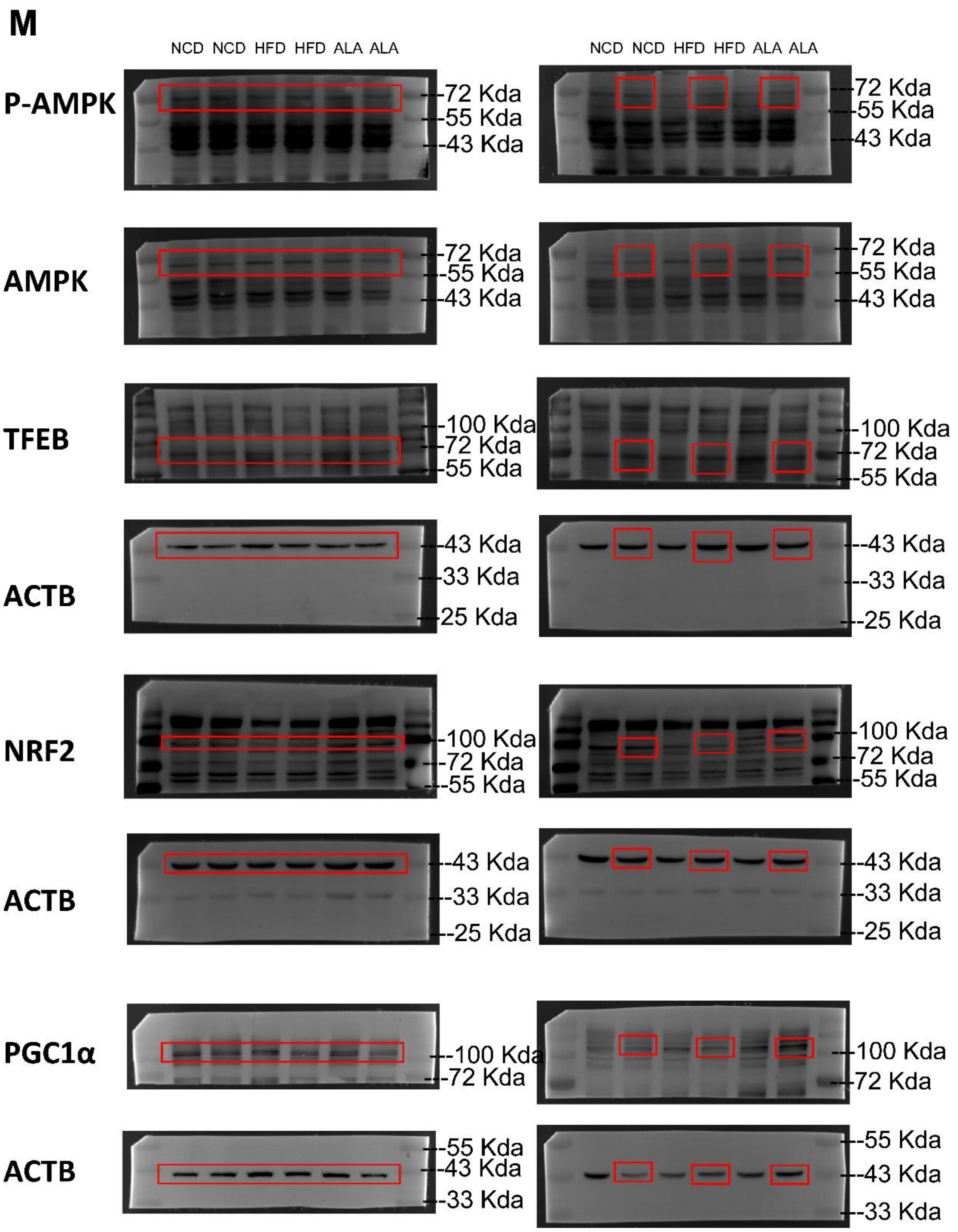

Supplemental Information

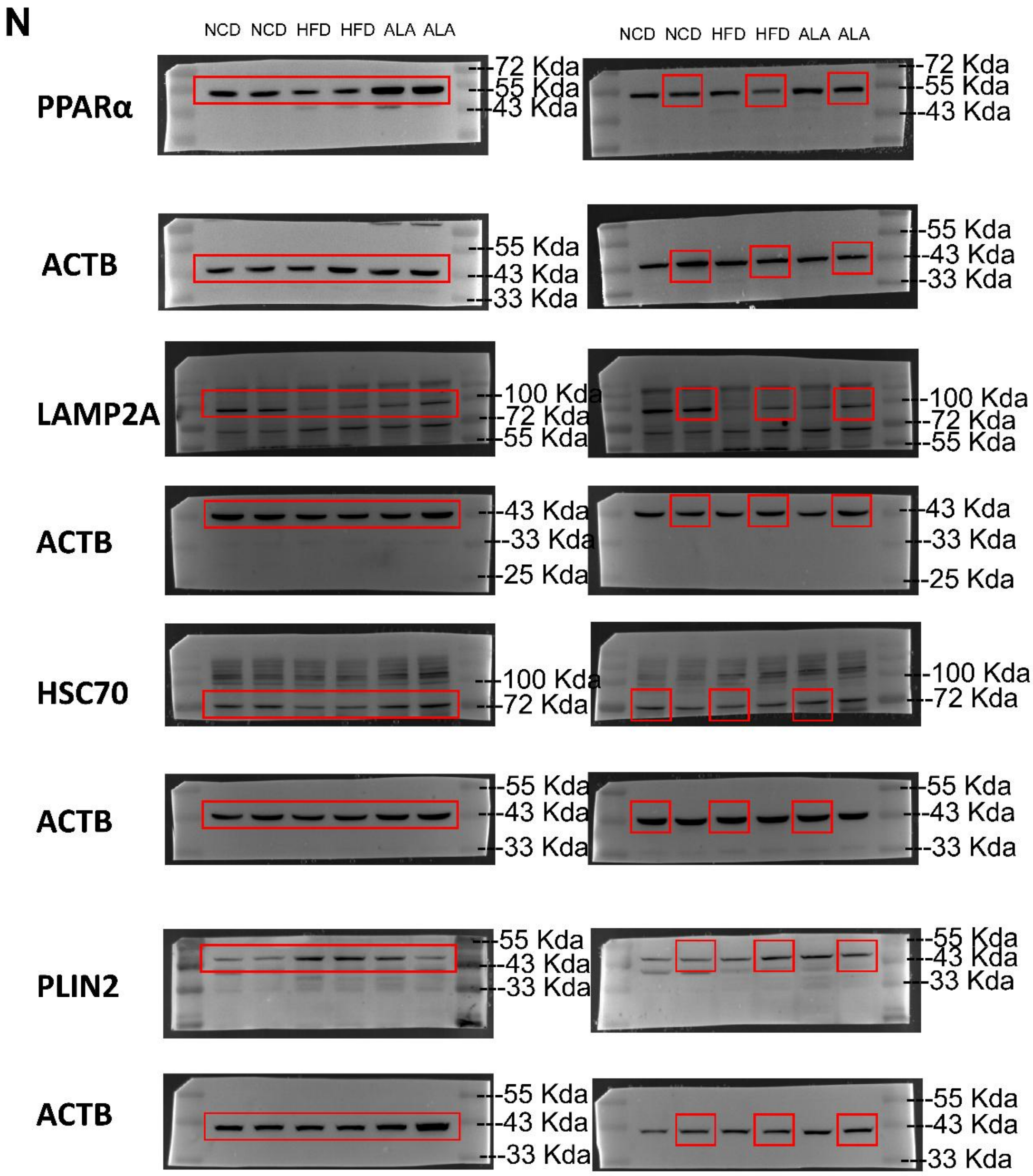

Figure S 7 Full-size images of western blots of liver. As shown in figure M and Figure N, mice were divided into three components, NCD, HFD and ALA, showing the full-size western blot images of animal liver tissue. Here is the expression level of p-AMPK, AMPK, TFEB, NRF2, PGC1α, PPARα, LAMP2A, HSC70 and PLIN2 in liver tissue. The red boxes indicate the cropped regions.

In the process of electrical transfer of proteins, multiple proteins were transfected from different parts of the same SDS-PAGE gel using pre-stained protein marker as reference. In this process, we used membrane regeneration solution to incubate different

antibodies in PVDF membrane again. We have provided three independent repeats and submitted the whole western blot picture as an attachment.
